# Valorization of Agri-Food Industry Waste as a Source of Phytohormones and Evaluation of Its Biostimulatory Potential in Oat (*Avena sativa* L.) Cultivation

**DOI:** 10.3390/ma19194130

**Published:** 2026-09-27

**Authors:** Aneta Sienkiewicz, Małgorzata Kowczyk-Sadowy, Sławomir Obidziński, Paweł Cwalina, Alicja Piotrowska-Niczyporuk, Andrzej Bajguz, Aneta Adamczuk, Iwona Ciereszko, Ilona Kulus

**Affiliations:** 1 Department of Agri-Food Engineering and Environmental Management, Faculty of Civil Engineering and Environmental Sciences, Bialystok University of Technology, Wiejska 45E, 15-351 Białystok, Poland; m.kowczyk@pb.edu.pl (M.K.-S.); s.obidzinski@pb.edu.pl (S.O.); p.cwalina@pb.edu.pl (P.C.); 2 Department of Biology and Plant Ecology, Faculty of Biology, University of Bialystok, Ciołkowskiego 1J, 15-245 Białystok, Poland; alicjap@uwb.edu.pl (A.P.-N.); abajguz@uwb.edu.pl (A.B.); a.adamczuk@uwb.edu.pl (A.A.); icier@uwb.edu.pl (I.C.); 3 Doctoral School, University of Bialystok, Ciołkowskiego 1J, 15-245 Białystok, Poland; i.kulus@uwb.edu.pl

**Keywords:** plant growth substances, post-production residues, agri-food waste, lemon balm, biostimulants, waste valorization

## Abstract

The valorization of agri-food industry waste is an important approach to obtaining value-added materials for agricultural applications while supporting the principles of a circular economy. Such waste may contain valuable nutrients and biologically active compounds, including plant hormones. This study aimed to evaluate the phytohormone content of post-production residues from the agri-food industry and to assess the biostimulatory potential of the material with the highest total phytohormone concentration that was also available in sufficient quantities as a post-production residue in oat (*Avena sativa* L.) cultivation. Phytohormones were determined in 37 samples using liquid chromatography–mass spectrometry (LC–MS) analysis. A total of 35 compounds representing six groups of plant hormones were identified: auxins, cytokinins, gibberellins, brassinosteroids, abscisic acid, and salicylic acid. Five samples—apple pomace, senna leaves, sage leaves, chicory roots, and tobacco stalks—contained all 35 compounds, whereas coffee grounds contained only eight. Brassinosteroids and auxins were the predominant hormone groups. Lemon balm leaves exhibited the highest total phytohormone concentration (511.26 ng·g^−1^ dry weight), while coffee grounds had the lowest (0.05 ng·g^−1^ dry weight). Dried and ground lemon balm leaves were therefore selected for a pot experiment conducted under controlled phytotron conditions. High proportions of lemon balm leaf waste reduced shoot growth, biomass accumulation, relative chlorophyll content, and nitrogen balance index (NBI), while increasing flavonoid and anthocyanin accumulation. In contrast, lower amendment promoted biomass production and maintained physiological and photosynthetic performance at levels comparable to or higher than those of the control plants. Most chlorophyll *a* fluorescence parameters remained relatively stable, although PI_ABS_ decreased at the highest amendment proportions. These findings indicate that lemon balm tea-processing waste may represent a potential source of biologically active compounds for agricultural applications. Its use at appropriate proportions may contribute to the valorization of plant-derived waste while supporting sustainable crop production and the principles of a circular economy.

## 1. Introduction

The continuous global expansion of the population has led to increased pressure on agriculture and food supply. Consequently, there is a pressing need to develop organic biostimulants that improve crop yield and soil quality without adversely affecting the environment or human health. In the United States, the 2018 Farm Bill [1] defines biostimulants as substances that, when applied directly to plants, seeds, or soil, induce beneficial effects on yield or growth. In Europe, under European Regulation 2019/1009 [2], biostimulants are described as fertilizing products that stimulate plant nutrition processes independent of the product’s nutrient content. This stimulation improves one or more characteristics of the plant or its rhizosphere, such as nutrient use efficiency, tolerance to abiotic stress, crop quality traits, or the availability of confined nutrients. Research indicates that the application of organic biostimulants, even in low concentrations, can enhance plant growth and development. This finding is supported by Sharma et al. [3], who noted improvements in plant development, and by Abdel-Raouf et al. [4], who observed enhancements in the chemical, physical, and biological properties of soil. These natural materials contain high-value compounds and active plant growth substances, such as phytohormones [5]. Key regulatory hormones in plant growth and development include auxins (AXs), brassinosteroids (BRs), cytokinins (CKs), gibberellins (GAs), abscisic acid (ABA), and salicylic acid (SA), which have been extensively studied [6,7,8].

The Environmental Protection Agency (EPA) reports that 45 billion kg of fresh vegetables, fruits, milk, and grain products become waste each year in the United States, costing about $1 billion annually to dispose of. In the United Kingdom, approximately twenty million tons of waste are generated, mainly from the fruit and vegetable/olive oil, fermentation, dairy, meat, and seafood industries [9]. In Poland, the agri-food industry primarily produces organic residues of plant and animal origin, characterized by specific fertilizer, energy, and feed properties [10]. These residues are rich in nutrients and bioactive components [11]. For example, herbal waste from herbal enterprises, such as peppermint, sage, chamomile, and nettle, is generated during herb processing, sorting, drying, packaging, and the production of food, medicinal, and cosmetic preparations. These wastes contain fatty acid methyl esters (FAMEs) and can potentially be used for biodiesel production [12]. Similarly, waste products from fruit and vegetable processing, like peels, seeds, pomace, and pulp, can be sources of phytochemicals and antioxidants. Goulas and Manganaris [13] observed that fruit peels contain higher amounts of phenolic compounds and ascorbic acid compared to the flesh, especially in unripe fruits. Additionally, due to their high carbon and nitrogen content and low levels of heavy metals, vegetable and fruit pomaces are suitable for biogas production [14].

Phytohormones are crucial in agriculture, promoting processes like fruit ripening, seed germination, and increasing crop yields [15,16]. Subbaiya et al. [17] demonstrated the effectiveness of a medium with vegetable waste for plant tissue culture in large-scale plant production. Kang et al. [18] found that organic fertilizer containing food waste positively influenced cabbage seedling growth. Whey, a by-product of the dairy industry, is utilized for producing various components including plant hormones [19]. Chóez et al. [20] discovered that maize waste extract, rich in *trans*-zeatin, could be used as a biostimulant. Similarly, Donno et al. [21] found that extracts from apple seeds, rapeseed, and rice husk waste enhanced kiwi fruit weight due to their auxin, gibberellin, and cytokinin content. Al-Maliki and Al-Masoudi [22] also emphasized the positive impact of phytohormones on crop productivity when treated with biostimulants. Taken together, these studies demonstrate that plant- and food-derived residues can contain phytohormones and other biologically active compounds that may contribute to their potential as plant growth-promoting materials.

It is known that domestic and agricultural organic wastes contain plant hormones [23,24]. However, little attention has been given to identifying these hormones in waste from the agri-food industry. Thus, the current study aims to evaluate the content of plant hormones such as AXs, BRs, CKs, GAs, ABA, and SA in agricultural and food processing waste, using liquid chromatography-mass spectrometry (LC-MS) analysis and to provide a comparative assessment of their phytohormonal profiles. Based on the obtained results, the material with the highest total phytohormone concentration and sufficient availability as a post-production residue was selected to assess its biostimulatory potential in oat (*Avena sativa* L.) cultivation.

## 2. Materials and Methods

### 2.1. Agri-Food Waste

Samples of 37 types of agri-food waste were collected from manufacturers processing natural plant materials in the Podlaskie Voivodeship, Poland, including Herbapol-Lublin S.A. (Białystok Branch, Białystok, Poland) and Kuchnia Vikinga (Białystok, Poland). These samples encompassed a diverse range of plant parts, including leaves (such as senna, St. John’s wort, sage, nettle, peppermint, herbal tea, green tea, lemon balm, and grass), flowers (chamomile), barks (buckthorn), roots (chicory and couch grass), and stalks (tobacco, black cumin, hemp). Also included were husks and shells (from onion, peanut, walnut, buckwheat, and rye), seeds (pepper, cherry, plum, quince, pumpkin, peach, melon, grape, watermelon, apricot, lemon, avocado, and apple), as well as various residues like tea grounds, coffee grounds, and apple pomace.

### 2.2. Chemicals

All chemicals used for phytohormone extraction, including salicylic acid (SA), were procured from Merck KGaA (Darmstadt, Germany). The solvents employed in liquid chromatography-mass spectrometry (LC-MS) analyses were of high-performance grade. A comprehensive range of phytohormone standards was purchased from OlChemIm (Olomouc, Czech Republic), i.e., abscisic acid (ABA), indole-3-acetic acid (IAA), indole-3-butyric acid (IBA), indole-3-pyruvic acid (IPA), phenylacetic acid (PAA), brassinolide (BL), 28-homobrassinolide (HBL), 24-epibrassinolide (epiBL), 28-norbrassinolide (norBL), 24-epicastasterone (epiCS), cathasterone (CT), 6-deoxytyphasterol (6dTY), *cis*-zeatin (cZ), *cis*-zeatin-riboside (cZR), *cis*-zeatin-9-glucoside (cZ9G), *cis*-zeatin-*O*-glucoside riboside (cZOGR), *trans*-zeatin (tZ), *trans*-zeatin-riboside (tZR), *trans*-zeatin-*O*-glucoside (tZOG), *trans*-zeatin-*O*-glucoside riboside (tZOGR), *trans*-zeatin-9-glucoside (tZ9G), *trans*-zeatin-9-glucoside-*O*-glucoside (tZ9GOG), dihydrozeatin (DHZ), dihydrozeatin riboside (DHZR), dihydrozeatin-*O*-glucoside (DHZOG), dihydrozeatin-*O*-glucoside riboside (DHZOGR), dihydrozeatin-7-glucoside (DHZ7G), dihydrozeatin-9-glucoside (DHZ9G), *N*^6^-isopentenyladenine (IP), *N*^6^-isopentenyladenosine-7-glucoside (IPR7G), *o*-topolin (oT), *m*-topolin (mT), *p*-topolin (pT), benzyladenine (BA), and gibberellic acid (GA_3_).

### 2.3. Phytohormone Extraction

For the measurement of phytohormones, 500 mg of powder was placed into each 2 mL Eppendorf tube. The powder was suspended in 1 mL of 50% (*v*/*v*) acetonitrile (ACN) and homogenized using a bead mill homogenizer (OMNI International, a PerkinElmer company, Kennesaw, GA, USA) at 3.1 m·s^−1^ for 3 cycles of 3 min each, utilizing two 3 mm tungsten balls. Following this, the samples were further homogenized using an ultrasound processor VCX 130 (Sonics & Materials Inc., Newtown, CT, USA) set to a power of 130 W and a frequency of 20 kHz for 5 min. This was done with a titanium probe and then mixed in a laboratory shaker (LC-350, Pol-Eko-Aparatura, Wodzisław Śląski, Poland) at 90 rpm in the dark at 5 °C for 30 min. After shaking, the samples were centrifuged at 9000× *g* for 5 min using an MPW-55 centrifuge (Med. Instruments, Gliwice, Poland) and the supernatant was collected in a glass tube. For quantification of phytohormones, stable isotope-labeled internal standards, such as [^2^H_6_] (+)-*cis*, *trans*-ABA (50 ng), [^2^H_5_] IAA (30 ng), [^2^H_6_] IP (50 ng), [^2^H_5_] tZ (30 ng), [^2^H_5_] tZOG (30 ng), [^2^H_3_] DHZR (30 ng), [^2^H_2_] GA_3_ (30 ng), [^2^H_3_] BL (20 ng) and [^2^H_3_] CS (20 ng), were added to the samples in specified quantities. The extracts were then prepared using a Waters SPE Oasis^®^ HLB cartridge (Waters Corporation, Milford, MA, USA), which was activated and equilibrated with 1 mL of 100% methanol (MeOH), 1 mL of H_2_O, and 1 mL of 50% (*v*/*v*) ACN. The extracts were loaded onto the cartridge, collected in Eppendorf tubes, and eluted with 1 mL of 30% (*v*/*v*) ACN. The samples were then evaporated to dryness using a Labconco CentriVap micro IR centrifugal vacuum concentrator (Labconco Corp., Kansas City, MO, USA) and reconstituted in 50 μL of 30% (*v*/*v*) ACN before being transferred to insert vials for analysis [25].

### 2.4. LC-MS Analysis of Phytohormones

Targeted compounds were analyzed using an LC-MS 8050 system (Shimadzu Corporation, Kyoto, Japan) comprising a pump, degasser, autosampler, column oven, and a triple quadrupole mass spectrometer. We injected 10 μL of each sample into a Waters XSelect C18 column (250 mm × 3.0 mm, 5 μm, Waters Corporation, Milford, MA, USA), maintained at 50 °C. The mobile phase consisted of a binary gradient of 0.01% (*v*/*v*) formic acid (FA) in ACN (A) and 0.01% (*v*/*v*) aqueous FA (B), with a flow rate of 0.5 mL·min^−1^. The separation was performed in ESI positive mode using the following gradient: 0–8 min, linear increase from 5 to 30% A; 8–25 min, 80% A; 25–28 min, 100% A; 28–30 min, returning to 5% A. Analytical data were processed using Shimadzu BrowserWorkstation Software (version 5.6) for LC-MS (Shimadzu Corporation, Kyoto, Japan) [25].

To validate the LC-MS method, plant extracts were analyzed, and the endogenous levels of the analytes were subtracted from the amounts of non-labeled standards added. The calculated concentrations of each analyte were then compared with the known amounts added to the samples to determine the accuracy of the method. Method precision was calculated as the relative standard deviation (RSD) of analyte concentrations determined in three replicates. The average RSD values ranged from 1.08% for ABA, 2.81% for AXs, 4.05% for CKs, and 5.11% for SA to 8.15% for BRs. Furthermore, a reproducibility test was performed by reinjecting standard mixtures prepared at low, medium, and high concentrations of the targeted compounds, covering a four-order-of-magnitude concentration range. Solutions kept in the autosampler at 4 °C were analyzed in triplicate on three consecutive days. For all three concentration levels, the RSD values of the analyte responses were below 15%.

### 2.5. Plant Material and Growth Conditions

The experiment was conducted using seedlings of common oat (*Avena sativa* L.) cv. Wulkan, a cultivar registered in 2023. Seeds were obtained from Plant Breeding Strzelce Sp. z o.o., IHAR Group, Strzelce, Poland.

Seeds were germinated for seven days under controlled phytotron conditions. The resulting seedlings were then transplanted into pots containing universal horticultural soil (pH 5.5–6.5; H. Rudnik, Poland) supplemented with different proportions of a post-production powdered fraction generated during lemon balm leaf processing (Table 1). Three seedlings were planted per pot, and 4–6 replicate pots were prepared for each treatment in each cultivation experiment.

Plants were grown under controlled phytotron conditions with a 16 h light/8 h dark photoperiod, day/night temperatures of 23/18 °C, relative air humidity of 60%, and photosynthetically active radiation (PAR) of 150 µmol·m^−2^·s^−1^. Plant material was sampled after 1, 2, and 3 weeks of cultivation for morphological, biomass, and physiological analyses. The remaining plants were maintained for up to six weeks to monitor the onset and development of inflorescences (panicles).

### 2.6. Assessment of Growth Parameters and Productivity Indices

Plant growth was evaluated during the first three weeks of cultivation. Shoot height and root length were measured weekly (*n* = 11 plants per treatment). At each sampling time, shoots and roots were separated and weighed to determine fresh mass. The plant material was then dried for 24 h at 105 °C in a laboratory drying oven (UN110, Memmert, Schwabach, Germany) and reweighed to determine dry mass (*n* = 7 plants per treatment). Fresh and dry biomass measurements were performed using an analytical balance (E42 S, RADWAG, Radom, Poland).

Water Content (WC)—the relative water content of plant tissues was calculated separately for shoots and roots based on the difference between fresh and dry mass. In addition, selected growth and biomass allocation indices were calculated to characterize the response of oat seedlings to the applied post-production material. The following growth parameters were calculated: Plant Length Ratio (PLR)—the ratio of root length to shoot height; Shoot Weight Ratio (SWR)—the ratio of shoot dry mass to total plant dry mass, reflecting biomass allocation to the aboveground parts of the plant; and Relative Growth Rate (RGR)—the relative rate of biomass accumulation over the experimental period, as described in previous studies [26,27,28].

### 2.7. Non-Invasive Assessment of Leaf Biochemical Status

To complement the morphological and biomass measurements, non-invasive measurements of selected biochemical leaf parameters were performed weekly during the first three weeks of plant growth (*n* = 10 plants per treatment).

Relative chlorophyll, flavonoid, and anthocyanin contents were determined using a Dualex Scientific+ instrument (Force-A, Orsay, France). Based on the chlorophyll and flavonoid indices, the Nitrogen Balance Index (NBI) was calculated as the ratio of relative chlorophyll to flavonoid content and used as an indirect indicator of plant nitrogen status [29].

### 2.8. Chlorophyll a Fluorescence Measurements

The physiological response of oat seedlings was further evaluated by analyzing chlorophyll *a* fluorescence kinetics using a Pocket PEA fluorimeter (Hansatech Instruments Ltd., King’s Lynn, UK).

Prior to measurement, leaves (*n* = 8 plants per treatment) were dark-adapted for 30 min to allow relaxation of the photosynthetic apparatus and ensure comparable initial conditions, as described by Macioszek et al. [29]. Chlorophyll *a* fluorescence was then induced using actinic light with an intensity of 3500 µmol·m^−2^·s^−1^.

The resulting fluorescence transients were analyzed using PEA Plus software (version 1.10, Hansatech Instruments Ltd., King’s Lynn, UK). The recorded fluorescence parameters were used to characterize the functional status and performance of the photosynthetic apparatus in oat seedlings exposed to different proportions of the post-production lemon balm leaf powder.

### 2.9. Data Analysis of Phytohormones

Data reported here represent the means of three replicates (*n* = 3). The dataset was tested for normality using the Shapiro–Wilk test and for homogeneity of variances using Levene’s test. Upon confirming the normality of data and homogeneity of variances, all results were subjected to a one-way analysis of variance (ANOVA). The means were then differentiated using the Scott-Knott test in R statistical software (version 4.2.2) [30]. The significance level for all statistical tests was set at *p* ≤ 0.05.

## 3. Results and Discussion

### 3.1. Phytohormone Content

The studies we conducted revealed the presence of various groups of phytohormones in agri-food waste, including auxins (AXs), brassinosteroids (BRs), cytokinins (CKs), gibberellins (GAs), abscisic acid (ABA), and salicylic acid (SA), as shown in Table 2. The average content of these phytohormones ranged from 0.0009 to 62.6143 ng·g^−1^ dry weight (DW). Brassinosteroids and auxins were the most prevalent, while gibberellins were found in the smallest quantities.

Auxins and brassinosteroids, being major classes of growth-promoting hormones, dominated our findings. This is significant, as AXs are known to influence soybean growth, shoot architecture, nodulation, and yield [31], while BRs play a role in xylem differentiation in tomatoes [32] and maize root elongation [33]. Our study showed that different agri-food wastes exhibited varying ratios of AXs to BRs, as illustrated in Figure 1. Grape seeds had the highest level of BRs (98.30%), while lemon balm leaves had the lowest (8.07%). Conversely, lemon balm leaves had the highest proportion of AXs (91.74%), and melon seeds had the lowest (0.01%).

Gibberellins and abscisic acid were found in lower quantities in the tested waste (less than 3.50%). Plum seeds had the highest ABA content (3.23%), and tobacco stalks the lowest (0.002%). Black tea grounds had the highest proportion of GA_3_ (0.50%), while herbal tea had less than 0.0001%. Notably, black tea grounds, coffee grounds, and lemon seeds contained over 40% cytokinins. Buckwheat husk had the highest SA level (64.37%), followed by sage leaves (30.82%). Brassinosteroids in most vegetable and fruit seeds ranged from 59.40 to 98.30% (Figure 1). Our LC-MS analysis quantified up to thirty-five different phytohormones in the agri-food waste. Apple pomace, senna leaves, sage leaves, chicory roots, and tobacco stalks contained all 35 types, while coffee grounds contained only 8. Fewer than 30 types of phytohormones were identified in eight wastes, including black tea grounds, peach seeds, and several others. Jindo et al. [34] demonstrated a strong association between various levels of plant hormones and the morphological effects of humic substances in soil.

The highest total phytohormone concentration was found in lemon balm leaves (511.26 ng·g^−1^ DW), herbal tea (511.18 ng·g^−1^ DW), and tobacco stalks (360.94 ng·g^−1^ DW), as detailed in Table 3. Lemon balm leaves had the most auxins (469.07 ng·g^−1^ DW), while herbal tea had the highest brassinosteroid content (465.66 ng·g^−1^ DW). Gibberellins were the least abundant in all tested samples, ranging from 0.0001 to 0.0061 ng·g^−1^ DW. Black tea and coffee grounds had the lowest overall phytohormone content, with coffee grounds lacking abscisic acid, brassinosteroids, and gibberellins. The predominant phytohormones in waste from the agri-food industry were indole-3-acetic acid (IAA) and brassinolide (BL), with the highest concentrations found in lemon balm leaves, herbal tea, and tobacco stalks, each exceeding 50% (Tables S1–S3). The significance of IAA lies in its abundance and vital role in regulating gene expression, both upregulating and downregulating [35]. Kondhare et al. [36] proposed the function of auxin in the storage root development of various crops, including sweet potato and carrot. Arshad et al. [37] highlighted the role of IAA in enhancing wheat productivity. Similarly, BL, widely distributed in the plant kingdom, plays a key role in gene expression regulation [38]. Nakagawa et al. [39] reported BL’s significant impact on rice plant growth, particularly in stem elongation and root formation. Mardinata et al. [40] studied the interaction between BL and fig varieties, noting changes in growth and physiological aspects.

### 3.2. Effect of Lemon Balm Leaf Powder on Oat Growth and Biomass Production

After three weeks of cultivation, clear differences in plant growth and biomass production were observed among the experimental treatments (Tables S4–S6). The strongest growth inhibition occurred in W1, where mean shoot height was 20.17 cm, representing a 57.2% reduction compared with the control. Shoot and root dry masses were 0.073 and 0.039 g, respectively, corresponding to reductions of 82.7% and 69.5%.

Treatment W2 showed a moderate growth reduction, with shoot dry mass reaching approximately 50% of the control and shoot height being 22.8% lower. In contrast, W3–W5 showed growth parameters comparable to or higher than the control. In W4 and W5, shoot dry mass reached 0.522 and 0.528 g, respectively, corresponding to an increase of approximately 23–25% compared with the control, while shoot height remained at a comparable level.

Differences among treatments were also observed in inflorescence development during the sixth week of cultivation. The mean total number of inflorescences increased from 19% on the first observation day to 95% on the fourth observation day (Table S7).

Overall, oat growth showed a clear response to the proportion of LBLP applied. The highest proportion used in W1 markedly inhibited plant growth and biomass accumulation, whereas lower proportions, particularly those applied in W4 and W5, did not adversely affect growth and were associated with greater shoot biomass than in the control.

This response is consistent with previous reports showing that materials derived from industrial by-products may contain biologically active compounds capable of affecting plant growth and development, with the magnitude and direction of the response depending on both material properties and application rate [41]. In the present study, the observed responses may be partly associated with the phytohormonal composition of LBLP; however, this interpretation remains tentative, as other potentially influential factors, including phytotoxic effects, phenolic compounds, and microbial activity, were not investigated.

### 3.3. Relative Growth Rate

The lowest Relative Growth Rate (RGR) values for both shoots and roots were recorded in W1 (Table S5). Shoot and root RGR values were 0.082 and 0.028, respectively, corresponding to reductions of 52% and 68% compared with the control.

In contrast, treatments W3–W5 showed RGR values comparable to or slightly higher than those of the control. When considered together with the higher shoot dry mass recorded in W4 and W5, these findings suggest that lower proportions of LBLP may favor biomass accumulation during the early stages of oat development.

This observation is consistent with the findings of Liu et al. [42], who identified whole-plant RGR as an important indicator of early vigor and demonstrated its association with leaf area productivity and plant nitrogen productivity in spring wheat.

### 3.4. Biomass Allocation

The Shoot Weight Ratio (SWR) and root/shoot ratio (PLR) revealed differences in biomass allocation between aboveground and belowground plant organs (Tables S4 and S6). The highest PLR value was recorded in W1 (0.867), whereas the lowest was recorded in W5 (0.365). This shift primarily resulted from the pronounced reduction in root dry mass rather than from an increase in shoot biomass. A higher root-to-shoot biomass ratio does not necessarily result from the action of auxins; rather, it may be associated with stress conditions, such as limited mineral nutrient availability in the substrate.

In W3–W5, SWR values were similar to those of the control, indicating that lower LBLP proportions did not substantially affect biomass allocation between shoots and roots. Changes in the SWR values compared with the control at the third week of plant growth may be attributable to the action of auxins and/or other compounds present in LBLP. This interpretation is consistent with the use of SWR as an indicator of relative biomass allocation between aboveground and belowground plant organs [43].

### 3.5. Chlorophyll Content and Nitrogen Balance Index

Relative chlorophyll content varied among treatments and sampling dates (Figure 2). The highest values in the third week were recorded in the control, W4, and W5, whereas W1 consistently showed the lowest values and no clear increase over time.

The Nitrogen Balance Index (NBI) followed a similar pattern (Figure 3). The highest values were observed in the control, W4, and W5, while W1 consistently showed the lowest NBI. The reduced NBI values recorded in W1 may indicate a less favorable nitrogen-related physiological status, as this index has been widely used as a non-destructive proxy for plant nitrogen status [44].

### 3.6. Flavonoid Content

The highest relative flavonoid content was recorded in W1, while elevated values were also observed in W2 (Figure 4). In the remaining treatments, flavonoid levels were generally lower and comparable to those of the control.

The increased flavonoid accumulation observed in W1 and W2 coincided with reduced plant growth and lower relative chlorophyll content. This pattern may reflect the activation of protective metabolic responses under less favorable growth conditions induced by higher LBLP proportions, as the accumulation of phenolic compounds, including flavonoids, is commonly associated with plant responses to environmental stress [45].

### 3.7. Anthocyanin Content

Relative anthocyanin content varied according to both treatment and cultivation time (Figure 5). Across all treatments, the highest values were generally recorded during the second week of cultivation, followed by a pronounced decrease in the third week.

The highest anthocyanin content was observed in W1, which also exhibited the strongest growth inhibition and the lowest relative chlorophyll and NBI values. The transient increase in anthocyanin accumulation may therefore reflect the activation of protective metabolic mechanisms in plants exposed to the highest LBLP proportion [46].

### 3.8. Chlorophyll a Fluorescence

Analysis of chlorophyll *a* fluorescence indicated that LBLP application had a relatively limited effect on most of the measured fluorescence parameters (Figure 6). Fm, Fv, Fv/Fm, and Fv/F0 remained generally similar among treatments, although Fm and Fv tended to decrease over the cultivation period.

The most pronounced differences were observed for PI_ABS_. In the control, PI_ABS_ gradually increased over time, whereas W1 exhibited distinctly lower values accompanied by a decreasing trend. Reduced PI_ABS_ values were also observed in W2, while W3–W5 generally remained comparable to the control.

ABS/RC showed relatively little variation among treatments, suggesting that the amount of absorbed excitation energy per active reaction center remained relatively stable. More pronounced differences were observed for DIo/RC, with the highest values recorded in W1, particularly during the second week of cultivation. This increase suggests enhanced dissipation of absorbed excitation energy as heat at the reaction-center level.

Changes in JIP-test parameters, including reduced PI_ABS_ and increased energy dissipation, have also been reported in plants exposed to stress conditions. For example, alterations in PI_ABS_ and DIo/RC were observed in maize subjected to cadmium stress, where impaired photosynthetic performance was accompanied by changes in excitation energy fluxes at the PSII reaction-center level [47].

Overall, W4 exhibited a chlorophyll fluorescence profile most similar to that of the control. When considered together with its relatively high shoot biomass and chlorophyll content, these findings indicate that the LBLP proportion used in W4 did not adversely affect photosynthetic performance and was among the most favorable treatments evaluated in the present study.

It should be noted that the present experiment was conducted under controlled growth conditions that differ from those encountered under field conditions. In particular, the relatively low PAR intensity, constant temperature, and absence of water deficit or biotic stress may have influenced the response of oat plants to LBLP. Therefore, the observed effects, including the beneficial responses recorded at lower LBLP proportions, should be interpreted with caution and should not be directly extrapolated to field conditions. Further experiments under variable environmental conditions, including fluctuations in temperature, light intensity, and water availability, as well as under relevant biotic stresses, are needed to determine whether the observed responses are maintained under field conditions.

## 4. Conclusions

- The analyzed agri-food industry residues showed substantial variation in both the qualitative and quantitative profiles of plant hormones, indicating that such by-products may constitute valuable sources of biologically active compounds.
- Brassinosteroids and auxins were the predominant phytohormone groups detected in the analyzed materials, accounting for a substantial proportion of the identified phytohormonal compounds.
- Apple pomace, senna leaves, sage leaves, chicory roots, and tobacco stalks exhibited the broadest phytohormone profiles, as all 35 identified compounds were detected in these materials.
- Lemon balm leaves contained the highest total phytohormone concentration among the analyzed residues and were therefore selected for evaluation in oat cultivation.
- The response of oat plants to lemon balm leaf powder (LBLP) was clearly dependent on the application proportion. High LBLP proportions markedly reduced shoot and root growth, biomass accumulation, relative chlorophyll content, nitrogen balance, and photosynthetic performance.
- In contrast, lower LBLP proportions, particularly those used in treatments W4 and W5, maintained plant growth and physiological performance at levels comparable to or higher than those of the control and promoted greater shoot biomass accumulation.
- The increased flavonoid and anthocyanin contents observed at higher LBLP proportions may indicate the involvement of protective metabolic responses associated with unfavorable growth conditions.
- Chlorophyll *a* fluorescence parameters were generally stable at lower LBLP proportions, whereas the highest application level reduced PI_ABS_ and increased energy dissipation, indicating impaired photosynthetic performance under excessive amendment.
- Lemon balm tea-processing waste appears to be a potential source of biologically active compounds with biostimulatory potential; however, the observed plant responses strongly depended on the application rate. The absence of a conventional fertilizer or commercial biostimulant as a reference treatment limits direct comparison of the observed effects and warrants further investigation.
- The use of appropriately selected proportions of lemon balm leaf waste may support biomass production while contributing to the valorization of agri-food industry by-products and the implementation of circular economy principles in agriculture.
- Overall, the comparative dataset of phytohormonal profiles generated in this study may provide a useful basis for identifying and selecting agri-food industry residues with specific hormone profiles for further evaluation of their potential biostimulatory properties.

## Figures and Tables

**Figure 1:**
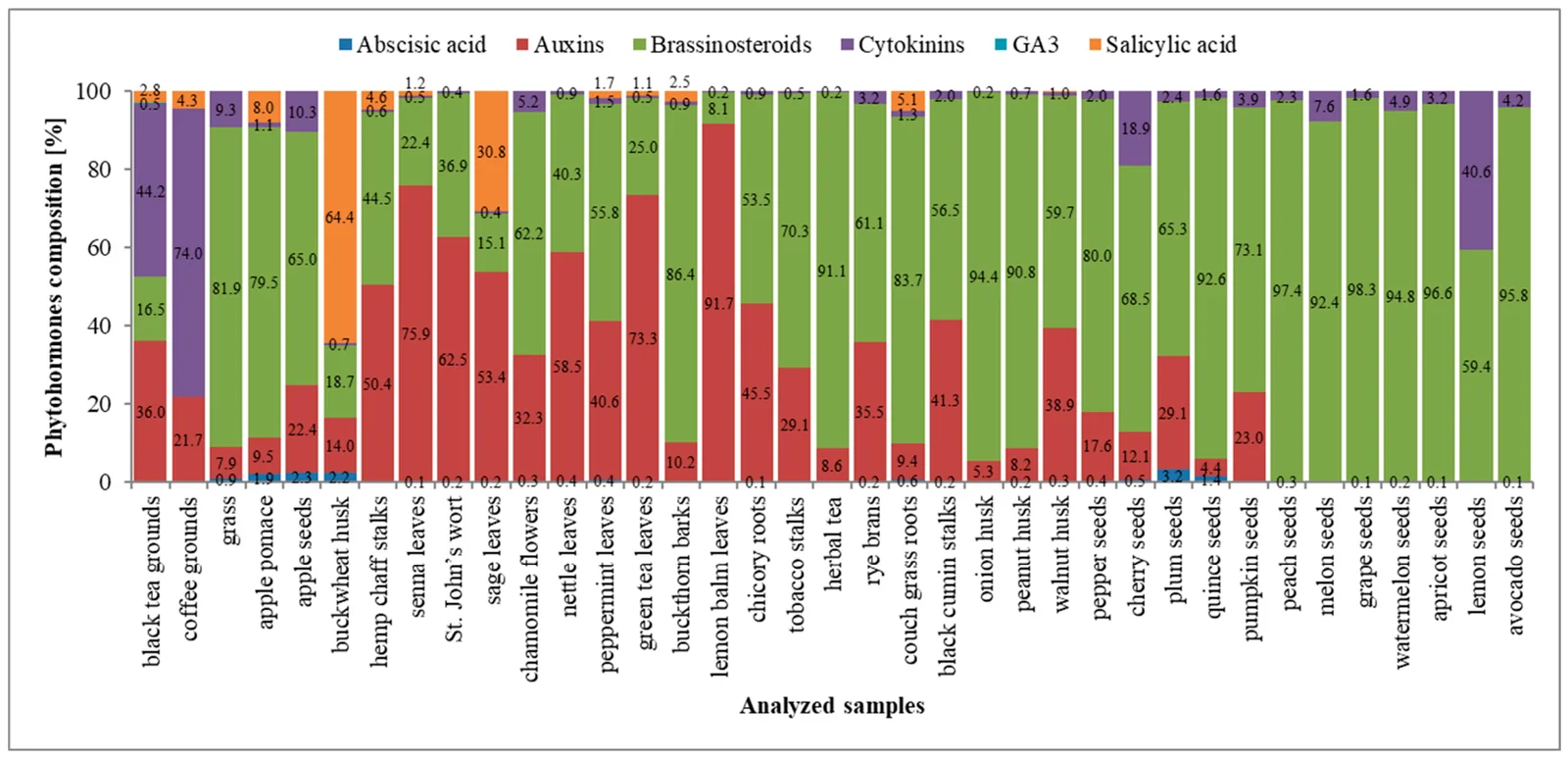
Phytohormones composition of analyzed wastes from the agricultural and food industry.

**Figure 2:**
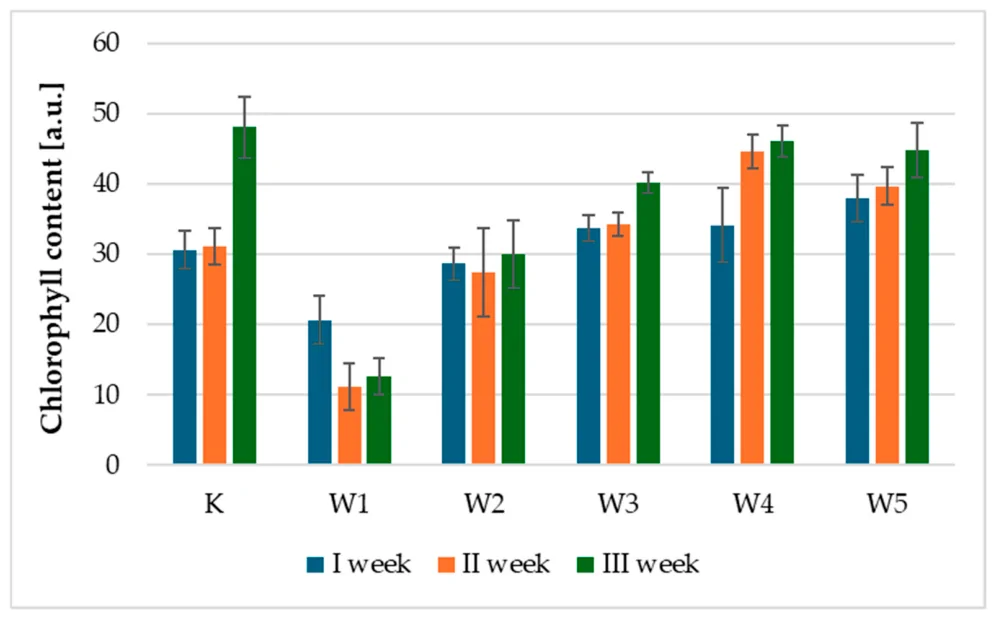
Relative chlorophyll content in oat leaves after 1, 2, and 3 weeks of cultivation under different experimental treatments. Values are expressed in arbitrary units (a.u.). Error bars represent variability of the measurements.

**Figure 3:**
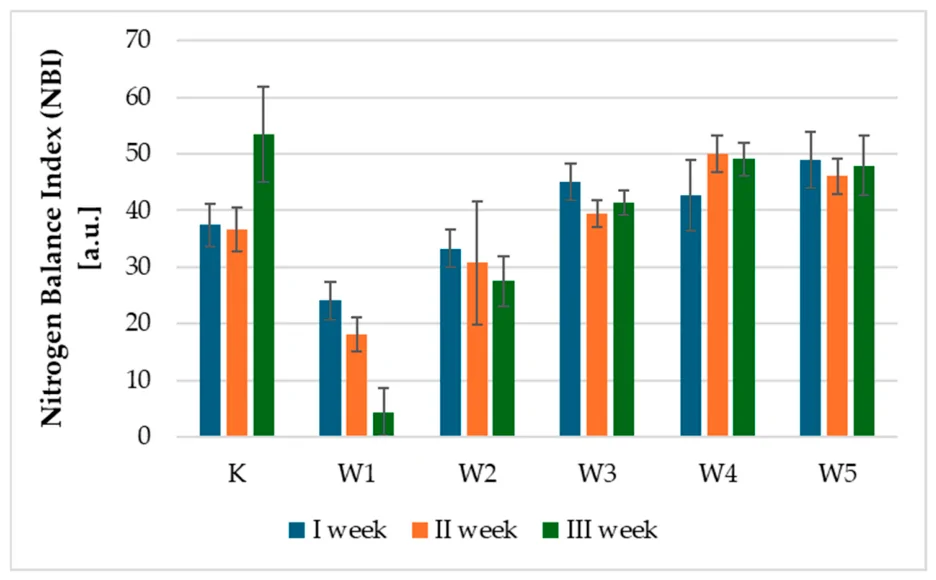
Nitrogen Balance Index (NBI) in oat leaves after 1, 2, and 3 weeks of cultivation under different experimental treatments. Values are expressed in arbitrary units (a.u.). Error bars represent variability of the measurements.

**Figure 4:**
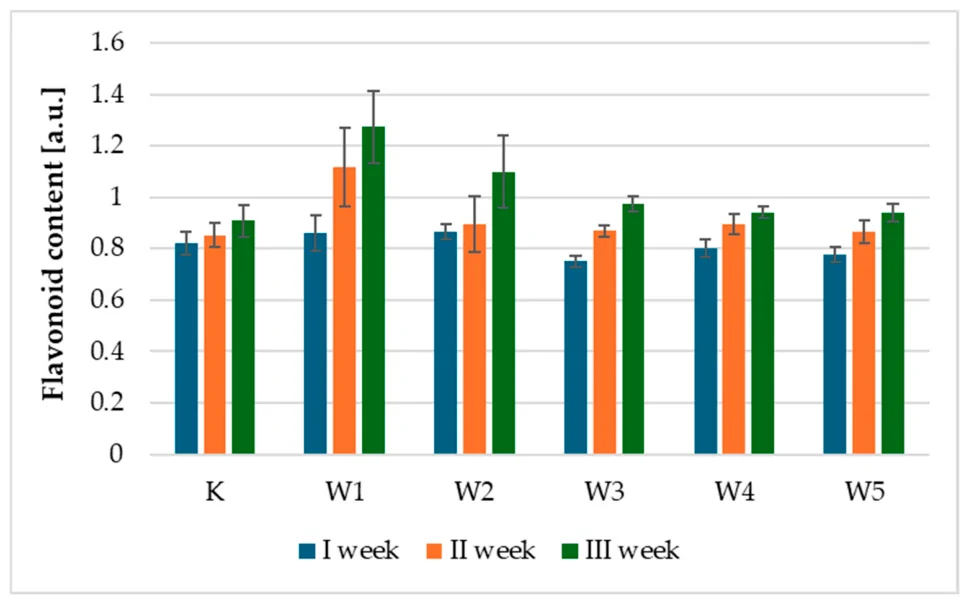
Flavonoid content in oat leaves after 1, 2, and 3 weeks of cultivation under different experimental treatments. Values are expressed in arbitrary units (a.u.). Error bars represent variability of the measurements.

**Figure 5:**
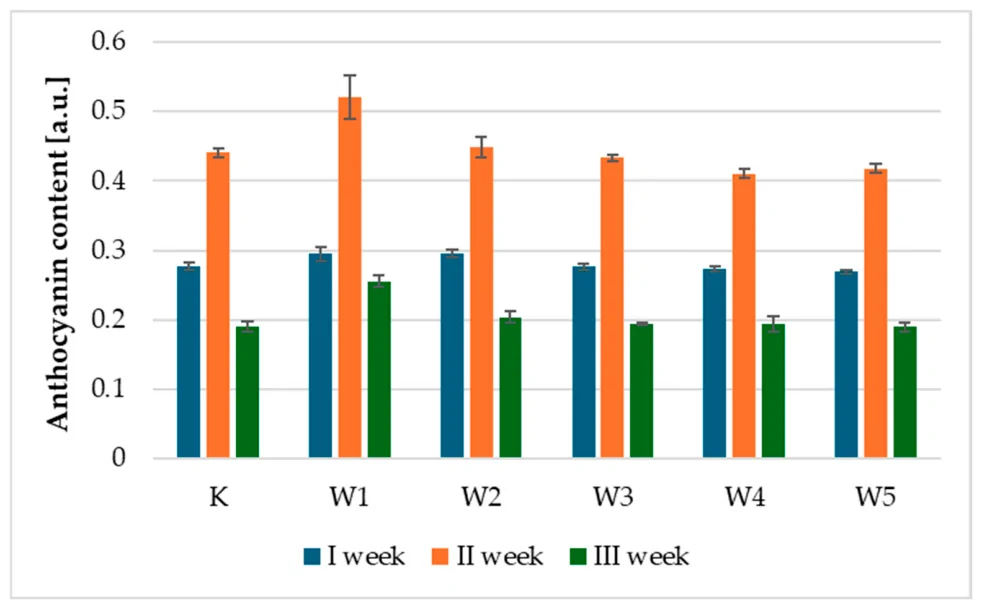
Anthocyanin content in oat leaves after 1, 2, and 3 weeks of cultivation under different experimental treatments. Values are expressed in arbitrary units (a.u.). Error bars represent variability of the measurements.

**Figure 6:**
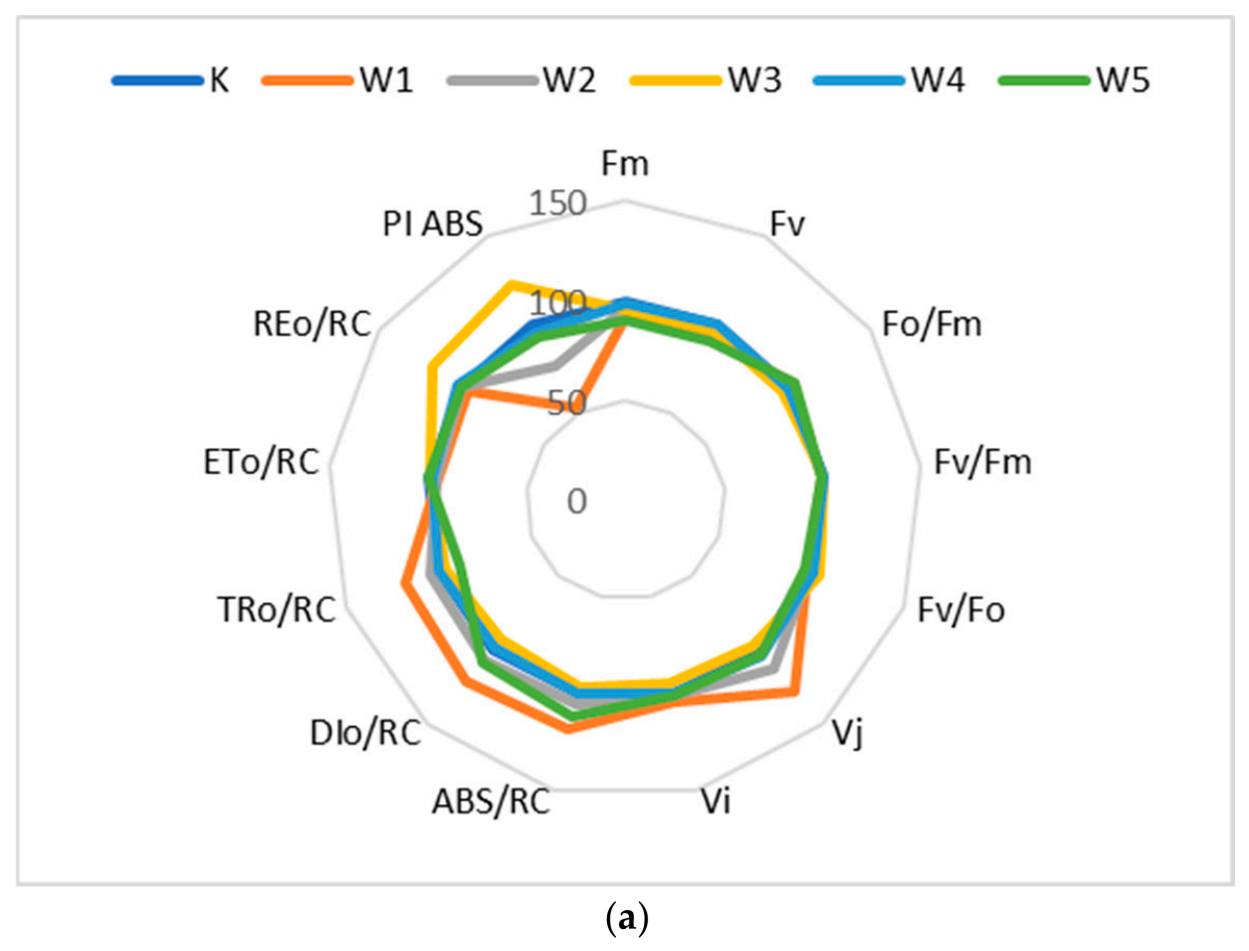

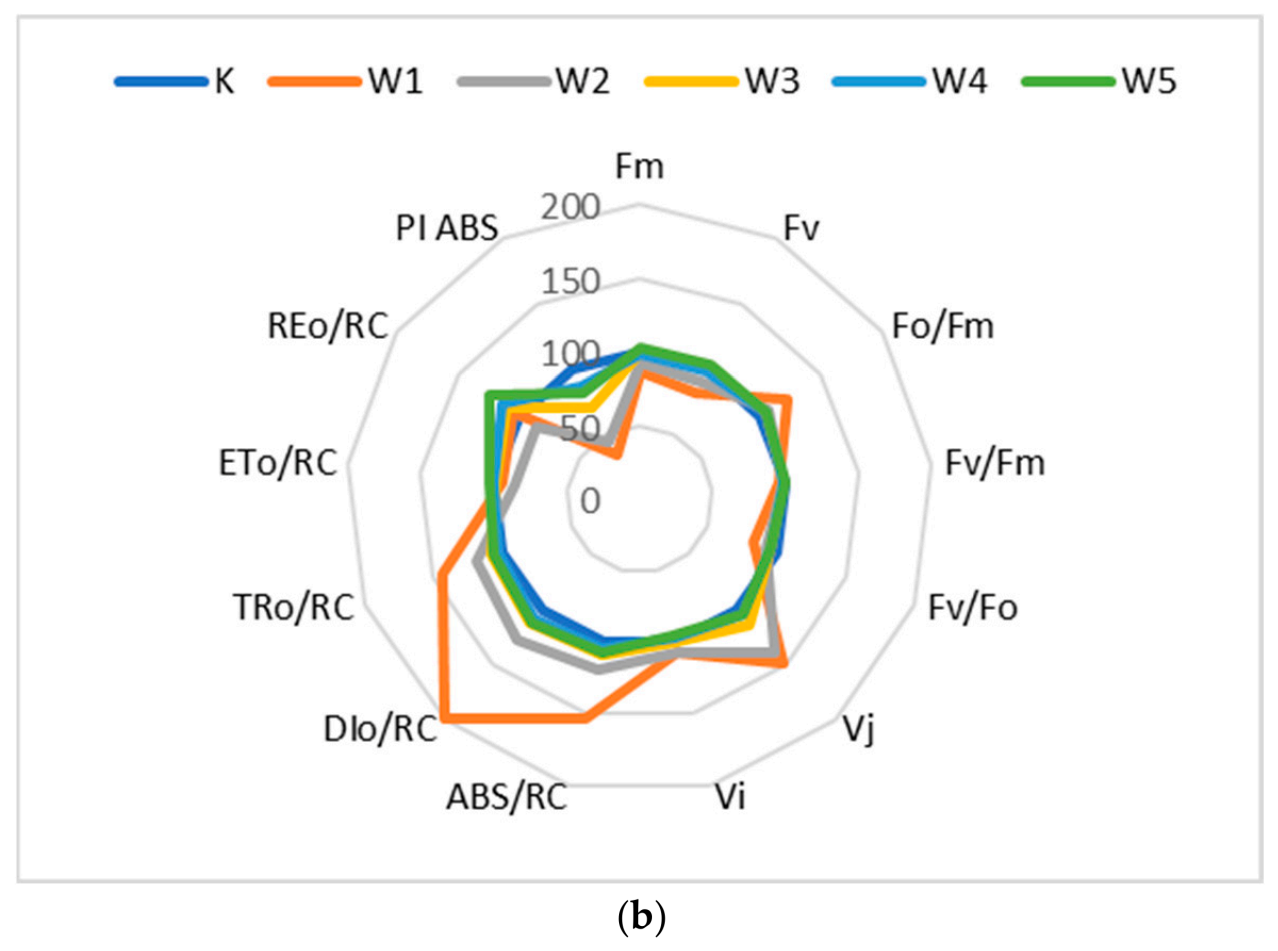

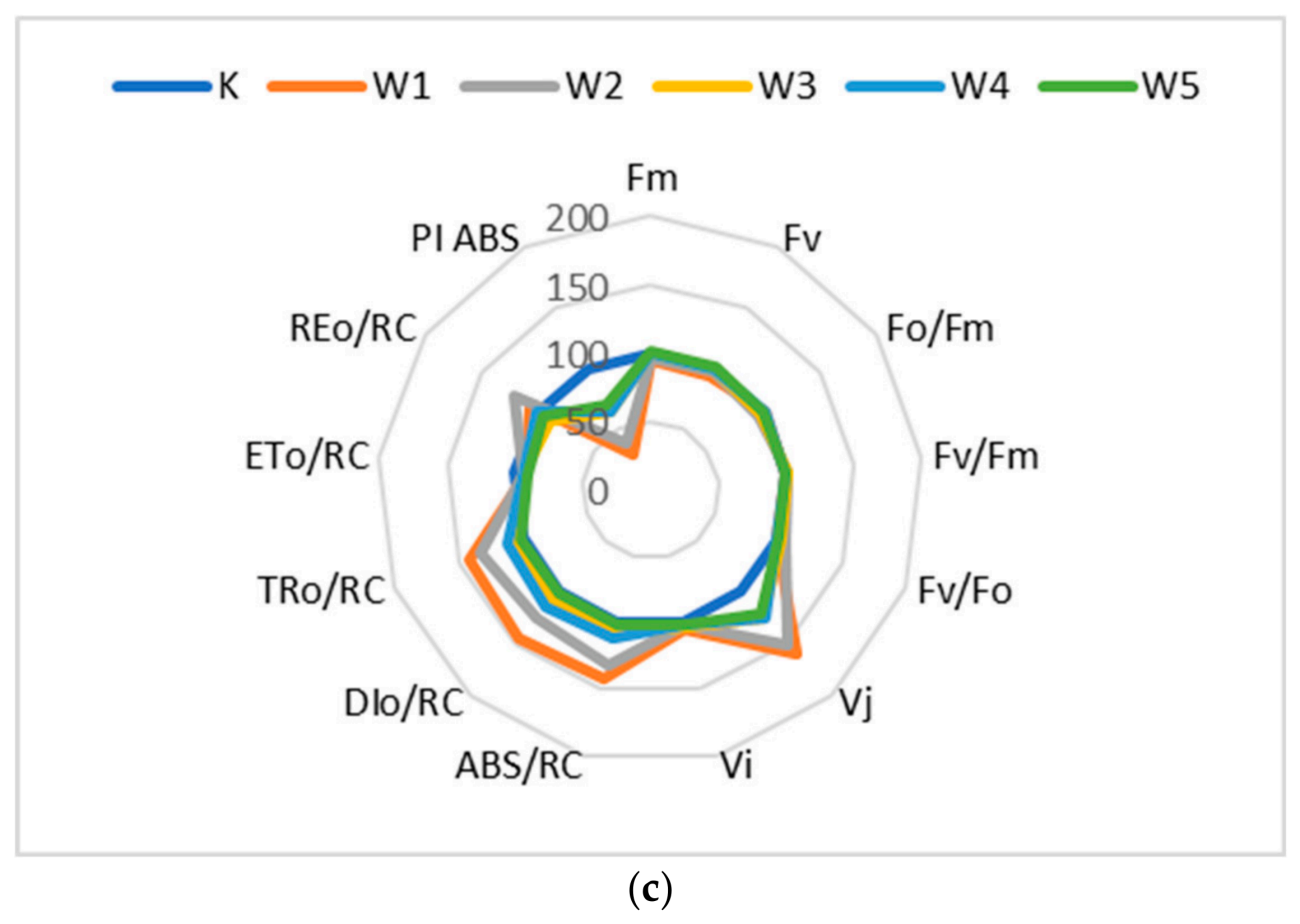
Values of selected chlorophyll *a* fluorescence parameters, expressed as a percentage of the control values, after 1 (**a**), 2 (**b**), and 3 (**c**) weeks of cultivation.

**Table 1:** Proportions of horticultural soil and lemon balm leaf powder (LBLP) used in the experimental treatments. The total mass of the soil–LBLP mixture in each pot was 100 g.

Treatment	Horticultural Soil [g]	Lemon Balm Leaf Powder [g]	Soil Ratio
K (control)	100.00	0.000	1:0
W1	50.000	50.000	1:1
W2	75.000	25.000	3:1
W3	87.500	12.500	7:1
W4	93.750	6.250	15:1
W5	96.875	3.125	31:1

**Table 2:** Average content and standard deviation of the studied groups of phytohormones in all tested waste.

Group of Phytohormones	Average Content	SD
	[ng·g^−1^ DW]	
Abscisic acid (ABA)	0.3698	0.0344
Auxins (AXs)	44.6261	3.9934
Brassinosteroids (BRs)	62.6143	3.9574
Cytokinins (CKs)	1.7156	0.1525
Gibberellic acid (GA_3_)	0.0009	0.0001
Salicylic acid (SA)	6.7539	0.1769

**Table 3:** Sum of mean phytohormones content ± standard deviation (SD) in the residues from agri-food processing both with and without distinction for abscisic acid, auxins, brassinosteroids, cytokinins, gibberellins and salicylic acid. ND, not detected.

Plant Materials	Abscisic Acid	Auxins	Brassinosteroids	Cytokinins	Gibberellins	Salicylic Acid	∑Phytohormones
[ng·g^−1^ DW]							
black tea grounds	ND	0.0509 ± 0.0114	0.0234 ± 0.0024	0.0625 ± 0.0021	<0.0100	<0.0100	0.1415 ± 0.0162
coffee grounds	ND	<0.0100	ND	0.0341 ± 0.0019	ND	<0.0100	0.0461 ± 0.0039
grass	0.4823 ± 0.0542	4.1024 ± 0.6834	42.6003 ± 5.8958	4.8286 ± 0.3030	<0.0100	ND	52.0197 ± 6.9367
apple pomace	1.3532 ± 0.1211	6.8101 ± 1.2435	56.8722 ± 2.7569	0.7627 ± 0.0618	<0.0100	5.7472 ± 0.2447	71.5470 ± 4.4282
apple seeds	1.0854 ± 0.1630	10.6615 ± 0.9368	30.8712 ± 2.6533	4.8707 ± 1.1127	<0.0100	ND	47.4904 ± 4.8659
buckwheat husk	1.4344 ± 0.0512	9.1585 ± 1.1677	12.2181 ± 1.6877	0.4326 ± 0.0518	<0.0100	41.9923 ± 1.4250	65.2371 ± 4.3836
hemp chaff stalks	0.0150 ± 0.0033	82.0727 ± 9.2733	72.4389 ± 3.3171	0.9311 ± 0.1457	<0.0100	7.4593 ± 0.8933	162.9180 ± 13.6328
senna leaves	0.1516 ± 0.0297	163.5049 ± 8.8212	48.2950 ± 4.4987	0.9920 ± 0.1200	<0.0100	2.5500 ± 0.0746	215.4939 ± 13.5446
St. John’s wort	0.4328 ± 0.0071	124.4680 ± 18.4293	73.4503 ± 6.0558	0.8672 ± 0.0886	<0.0100	ND	199.2191 ± 24.5809
sage leaves	0.5878 ± 0.0633	138.7553 ± 9.8731	39.3074 ± 4.0453	0.9332 ± 0.0693	<0.0100	80.0178 ± 0.6746	259.6035 ± 14.7259
chamomile flowers	0.2603 ± 0.0290	25.1213 ± 1.0986	48.4196 ± 2.6017	4.0126 ± 0.2315	<0.0100	ND	77.8157 ± 3.9609
nettle leaves	0.3003 ± 0.0366	49.0218 ± 1.5619	33.7803 ± 3.7630	0.7614 ± 0.0668	<0.0100	ND	83.8651 ± 5.4284
peppermint leaves	0.4267 ± 0.0279	40.0730 ± 1.1846	54.9874 ± 2.7069	1.4721 ± 0.2778	<0.0100	1.6511 ± 0.1027	98.6105 ± 4.3000
green tea leaves	0.2525 ± 0.2189	111.7830 ± 7.6861	38.0842 ± 1.3904	0.7557 ± 0.0442	<0.0100	1.6740 ± 0.0172	152.5496 ± 9.3569
buckthorn barks	ND	16.7709 ± 1.0575	142.1301 ± 7.6878	1.5569 ± 0.0761	<0.0100	4.1021 ± 0.5165	164.5602 ± 9.3380
lemon balm leaves	0.0587 ± 0.0011	469.0744 ± 55.2849	41.2743 ± 4.3221	0.8501 ± 0.0856	<0.0100	ND	511.2584 ± 59.6938
chicory roots	0.0421 ± 0.0052	14.4750 ± 2.6741	17.0159 ± 1.1642	0.2802 ± 0.0320	<0.0100	<0.0100	31.8185 ± 3.8761
tobacco stalks	<0.0100	105.0867 ± 4.8601	253.8656 ± 8.1753	1.9775 ± 0.3530	<0.0100	ND	360.9376 ± 13.3888
herbal tea	0.1769 ± 0.0044	44.1086 ± 3.6490	465.6608 ± 27.2465	1.2281 ± 0.1300	<0.0100	ND	511.1748 ± 31.0300
rye brans	0.1048 ± 0.0085	17.3280 ± 1.4470	29.7632 ± 2.4428	1.5509 ± 0.1152	<0.0100	<0.0100	48.7506 ± 4.0137
couch grass roots	1.0928 ± 0.0195	18.5838 ± 1.1157	165.6280 ± 7.4052	2.5163 ± 0.0609	<0.0100	10.0703 ± 0.1142	197.8923 ± 8.7156
black cumin stalks	0.2188 ± 0.0193	53.0039 ± 3.0083	72.5649 ± 3.4289	2.5555 ± 0.1696	<0.0100	ND	128.3440 ± 6.6262
onion husk	0.0244 ± 0.0008	6.2915 ± 0.8353	111.7888 ± 12.7128	0.2657 ± 0.0152	<0.0100	0.0123 ± 0.0021	118.3836 ± 13.5663
peanut husk	0.1589 ± 0.0072	5.2910 ± 0.1882	58.3139 ± 4.4027	0.4456 ± 0.0272	<0.0100	<0.0100	64.2113 ± 4.6258
walnut husk	0.2332 ± 0.0215	26.0195 ± 0.8307	39.8614 ± 0.8305	0.6578 ± 0.0280	<0.0100	0.0396 ± 0.0011	66.8122 ± 1.7119
pepper seeds	0.0631 ± 0.0044	3.0046 ± 0.4975	13.6295 ± 1.8475	0.3484 ± 0.0418	<0.0100	<0.0100	17.0460 ± 2.3914
cherry seeds	0.1660 ± 0.0031	3.7692 ± 0.7105	21.2606 ± 1.8468	5.8580 ± 0.4826	<0.0100	<0.0100	31.0579 ± 3.0436
plum seeds	0.3717 ± 0.0196	3.3426 ± 0.7474	7.5038 ± 0.5103	0.2793 ± 0.0780	<0.0100	<0.0100	11.4986 ± 1.3555
quince seeds	0.8419 ± 0.0431	2.5726 ± 0.0778	53.8321 ± 5.4124	0.9060 ± 0.1113	<0.0100	<0.0100	58.1541 ± 5.6450
pumpkin seeds	<0.0100	7.4456 ± 0.8028	23.6750 ± 0.5964	1.2773 ± 0.0998	<0.0100	<0.0100	32.4045 ± 1.4993
peach seeds	ND	0.0989 ± 0.0052	30.1781 ± 0.6425	0.7193 ± 0.0606	<0.0100	<0.0100	30.9975 ± 0.7085
melon seeds	ND	<0.0100	97.9693 ± 5.6839	8.0323 ± 0.7838	<0.0100	ND	106.0076 ± 6.4683
grape seeds	ND	<0.0100	14.8393 ± 1.7764	0.2439 ± 0.0145	<0.0100	ND	15.0967 ± 1.7917
watermelon seeds	ND	0.0165 ± 0.0018	7.5326 ± 0.6092	0.3929 ± 0.0263	<0.0100	ND	7.9424 ± 0.6374
apricot seeds	ND	0.0193 ± 0.0010	13.0672 ± 0.7582	0.4387 ± 0.0255	ND	ND	13.5252 ± 0.7847
lemon seeds	ND	ND	13.2060 ± 0.6801	9.0247 ± 0.2307	ND	ND	22.2307 ± 0.9108
avocado seeds	<0.0100	ND	8.2078 ± 0.9076	0.3560 ± 0.0165	<0.0100	<0.0100	8.5721 ± 0.9247

## Data Availability

The original contributions presented in this study are included in the article/Supplementary Materials. Further inquiries can be directed to the corresponding author.

## Supplementary Materials

The following supporting information can be downloaded at https://www.mdpi.com/article/10.3390/ma19194130/s1. Table S1. Average content of abscisic acid (ABA), salicylic acid (SA), gibberellic acid (GA3), auxins (IAA, IBA, IPA, PAA) ± standard deviation (SD) in residues from agricultural and food processing. ND, not detected. Table S2. Average content of brassinosteroids (BL, HBL, epiBL, norBL, epiCS, CT, 6dTY) ± standard deviation (SD) in residues from agricultural and food processing. ND, not detected. Table S3. Average content of cytokinins (cZ, cZR, cZ9G, cZOGR, tZ, tZR, tZOG, tZOGR, tZ9G, DHZ, DHZR, DHZOG, DHZOGR, DHZ7G, DHZ9G, IP, IPR7G, oT, mT, pT, BA) ± standard deviation (SD) in residues from agricultural and food processing. ND, not detected. Table S4. Shoot height, root length and root/shoot ratio (PLR) of oat plants during the first three weeks of cultivation under different experimental treatments (*n* = 11). Table S5. Shoot and root fresh and dry mass, Water Content (WC), and Relative Growth Rate (RGR) of oat plants during the first three weeks of cultivation under different experimental treatments (*n* = 7). Table S6. Shoot and root dry mass, and Shoot Weight Ratio (SWR) of oat plants during the first three weeks of cultivation under different experimental treatments (*n* = 7). Table S7. Percentage of oat inflorescences recorded over four consecutive days during the sixth week of cultivation (*n* = 9).

## Abbreviations

The following abbreviations are used in this manuscript:

ABA	abscisic acid
BA	benzyladenine
BL	brassinolide
CT	cathasterone
cZ	*cis*-zeatin
cZ9G	*cis*-zeatin-9-glucoside
cZOGR	*cis*-zeatin-*O*-glucoside riboside
cZR	*cis*-zeatin-riboside
6dTY	6-deoxytyphasterol
DHZ	dihydrozeatin
DHZ7G	dihydrozeatin-7-glucoside
DHZ9G	dihydrozeatin-9-glucoside
DHZOG	dihydrozeatin-*O*-glucoside
DHZOGR	dihydrozeatin-*O*-glucoside riboside
DHZR	dihydrozeatin riboside
epiBL	24-epibrassinolide
epiCS	24-epicastasterone
GA_3_	gibberellic acid
HBL	28-homobrassinolide
IAA	indole-3-acetic acid
IBA	indole-3-butyric acid
IP	*N*^6^-isopentenyladenine
IPA	indole-3-pyruvic acid
IPR7G	*N*^6^-isopentenyladenosine-7-glucoside
mT	*m*-topolin
norBL	28-norbrassinolide
oT	*o*-topolin
PAA	phenylacetic acid
pT	*p*-topolin
SA	salicylic acid
tZ	*trans*-zeatin
tZ9G	*trans*-zeatin-9-glucoside
tZ9GOG	*trans*-zeatin-9-glucoside-*O*-glucoside
tZOG	*trans*-zeatin-*O*-glucoside
tZOGR	*trans*-zeatin-*O*-glucoside riboside
tZR	*trans*-zeatin-riboside

## References

[1] (2018). Agriculture Improvement Act of 2018 (2018 Farm Bill). One Hundred Fifteenth Congress of the United States of America, Public Law 115–334. https://www.congress.gov/115/plaws/publ334/PLAW-115publ334.pdf.

[2] Regulation (EU) 2019/1009 of the European Parliament and of the Council of 5 June 2019 Laying down Rules on the Making Available on the Market of EU Fertilising Products and Amending Regulations (EC) No 1069/2009 and (EC) No 1107/2009 and Repealing Regulation (EC) No 2003/2003. https://eur-lex.europa.eu/eli/reg/2019/1009/oj/eng.

[3] Sharma H.S.S., Fleming C., Selby C., Rao J.R., Martin T. (2014). Plant Biostimulants: A Review on the Processing of Macroalgae and Use of Extracts for Crop Management to Reduce Abiotic and Biotic Stresses. J. Appl. Phycol..

[4] Abdel-Raouf N., Al-Homaidan A.A., Ibraheem I.B.M. (2012). Agricultural Importance of Algae. Afr. J. Biotechnol..

[5] Yakhin O.I., Lubyanov A.A., Yakhin I.A., Brown P.H. (2017). Biostimulants in Plant Science: A Global Perspective. Front. Plant Sci..

[6] Bajguz A., Chmur M., Gruszka D. (2020). Comprehensive Overview of the Brassinosteroid Biosynthesis Pathways: Substrates, Products, Inhibitors, and Connections. Front. Plant Sci..

[7] Chen K., Li G., Bressan R.A., Song C., Zhu J., Zhao Y. (2020). Abscisic Acid Dynamics, Signaling, and Functions in Plants. J. Integr. Plant Biol..

[8] Jamil M., Saher A., Javed S., Farooq Q., Shakir M., Zafar T., Komal L., Hussain K., Shabir A., Javed A. (2021). A Review on Potential Role of Auxins in Plants, Current Applications and Future Directions. J. Biodivers. Environ. Sci. JBES.

[9] Kosseva M.R. (2009). Processing of Food Wastes. Adv. Food Nutr. Res..

[10] Czyżyk F., Strzelczyk M. (2015). Rational Utilization of Production Residues Generated in Agri-Food. Arch. Waste Manag. Environ. Prot..

[11] Kowczyk-Sadowy M. (2021). Physicochemical and Biological Properties of Feed Granulates Produced from Agro-Food Processing Waste.

[12] Sienkiewicz A., Piotrowska-Niczyporuk A., Bajguz A. (2020). Fatty Acid Methyl Esters from the Herbal Industry Wastes as a Potential Feedstock for Biodiesel Production. Energies.

[13] Goulas V., Manganaris G.A. (2012). Exploring the Phytochemical Content and the Antioxidant Potential of Citrus Fruits Grown in Cyprus. Food Chem..

[14] Morales-Polo C., del Mar Cledera-Castro M., Moratilla Soria B.Y. (2019). Biogas Production from Vegetable and Fruit Markets Waste—Compositional and Batch Characterizations. Sustainability.

[15] Kang J., Yim S., Choi H., Kim A., Lee K.P., Lopez-Molina L., Martinoia E., Lee Y. (2015). Abscisic Acid Transporters Cooperate to Control Seed Germination. Nat. Commun..

[16] Lu Q., Zhang W., Gao J., Lu M., Zhang L., Li J. (2015). Simultaneous Determination of Plant Hormones in Peach Based on Dispersive Liquid–Liquid Microextraction Coupled with Liquid Chromatography–Ion Trap Mass Spectrometry. J. Chromatogr. B.

[17] Subbaiya R., Aakash B., Shanmugaraja A., Devika R., Chozhavendhan S., Vinoth S., Devi G.K., Selvam M.M. (2019). Vegetable Waste as an Alternate Plant Tissue Culture Media for Laboratory and Industry. Res. J. Pharm. Technol..

[18] Kang Y., Wu Q., Qin J., Zhong M., Yang X., Chai X. (2022). High Relative Humidity Improves Leaf Burn Resistance in Flowering Chinese Cabbage Seedlings Cultured in a Closed Plant Factory. PeerJ.

[19] George D.M., Vincent A.S., Mackey H.R. (2020). An Overview of Anoxygenic Phototrophic Bacteria and Their Applications in Environmental Biotechnology for Sustainable Resource Recovery. Biotechnol. Rep..

[20] Chóez I., Manzano Santana P., Peralta E. (2014). Quantification of Trans-Zeatin in Corn Wastes and Liquid Organic Fertilizers by HPLC Chromatography. Emir. J. Food Agric. EJFA.

[21] Donno D., Beccaro G.L., Mellano M.G., Canterino S., Cerutti A.K., Bounous G. (2013). Improving the Nutritional Value of Kiwifruit with the Application of Agroindustry Waste Extracts. J. Appl. Bot. Food Qual..

[22] Al-Maliki S., Al-Masoudi M. (2018). Interactions between Mycorrhizal Fungi, Tea Wastes, and Algal Biomass Affecting the Microbial Community, Soil Structure, and Alleviating of Salinity Stress in Corn Yield (*Zea mays* L.). Plants.

[23] Klimas E., Szymańska-Pulikowska A., Górka B.M., Wieczorek P. (2016). Presence of Plant Hormones in Composts Made from Organic Fraction of Municipal Solid Waste. J. Elem..

[24] Puglia D., Pezzolla D., Gigliotti G., Torre L., Bartucca M.L., Del Buono D. (2021). The Opportunity of Valorizing Agricultural Waste, through Its Conversion into Biostimulants, Biofertilizers, and Biopolymers. Sustainability.

[25] Sienkiewicz A., Krasowska M., Kowczyk-Sadowy M., Obidziński S., Piotrowska-Niczyporuk A., Bajguz A. (2024). Occurrence of Plant Hormones in Composts Made from Organic Fraction of Agri-Food Industry Waste. Sci. Rep..

[26] Rossi R., Bochicchio R., Labella R., Amato M., De Vita P. (2024). Phenotyping Seedling Root Biometry of Two Contrasting Bread Wheat Cultivars under Nutrient Deficiency and Drought Stress. Agronomy.

[27] Hoffmann W.A., Poorter H. (2002). Avoiding Bias in Calculations of Relative Growth Rate. Ann. Bot..

[28] Siemieniuk A., Ludynia M., Rudnicka M. (2021). Response of Two Crop Plants, *Zea mays* L. and *Solanum lycopersicum* L., to Diclofenac and Naproxen. Int. J. Mol. Sci..

[29] Macioszek V.K., Chalamońska K., Oliwa J., Staszak A.M., Sobczak M. (2025). Phenological, Physiological, and Ultrastructural Analyses of ‘Green Islands’ on Senescent Leaves of Norway Maple (*Acer platanoides* L.). Plants.

[30] R Core Team (2020). R: A Language and Environment for Statistical Computing.

[31] Roy Choudhury S., Johns S.M., Pandey S. (2019). A Convenient, Soil-free Method for the Production of Root Nodules in Soybean to Study the Effects of Exogenous Additives. Plant Direct.

[32] Lee J., Han S., Lee H.Y., Jeong B., Heo T.Y., Hyun T.K., Kim K., Je B.I., Lee H., Shim D. (2019). Brassinosteroids Facilitate Xylem Differentiation and Wood Formation in Tomato. Planta.

[33] Zhang H., Zhao D., Tang Z., Zhang Y., Zhang K., Dong J., Wang F. (2022). Exogenous Brassinosteroids Promotes Root Growth, Enhances Stress Tolerance, and Increases Yield in Maize. Plant Signal. Behav..

[34] Jindo K., Olivares F.L., da Paixão Malcher D.J., Sánchez-Monedero M.A., Kempenaar C., Canellas L.P. (2020). From Lab to Field: Role of Humic Substances under Open-Field and Greenhouse Conditions as Biostimulant and Biocontrol Agent. Front. Plant Sci..

[35] Sánchez-Parra B., Pérez-Alonso M.-M., Ortiz-García P., Moya-Cuevas J., Hentrich M., Pollmann S. (2021). Accumulation of the Auxin Precursor Indole-3-Acetamide Curtails Growth through the Repression of Ribosome-Biogenesis and Development-Related Transcriptional Networks. Int. J. Mol. Sci..

[36] Kondhare K.R., Kumar A., Patil N.S., Malankar N.N., Saha K., Banerjee A.K. (2021). Development of Aerial and Belowground Tubers in Potato Is Governed by Photoperiod and Epigenetic Mechanism. Plant Physiol..

[37] Arshad M.J., Arıf U., Nazeer A., Yasın G. (2022). Improvement of Wheat (*Triticum aestivum* L.) Productivity with the Applications of Plant Growth Regulators. Pak. J. Bot..

[38] Bajguz A., Hayat S., Yusuf M., Bhardwaj R., Bajguz A. (2019). Brassinosteroids in Microalgae: Application for Growth Improvement and Protection Against Abiotic Stresses. Brassinosteroids: Plant Growth and Development.

[39] Nakagawa Y., Nishikawa B., Miyagawa H. (2021). Effects of Brassinolide on the Growing of Rice Plants. J. Pestic. Sci..

[40] Mardinata Z., Mardaleni, Sabli T.E. (2021). Impact Brassinolide on Two Fig Varieties. Agroecosystems–Very Complex Environmental Systems.

[41] Gaidau C., Stanca M., Niculescu M.-D., Alexe C.-A., Becheritu M., Horoias R., Cioineag C., Râpă M., Stanculescu I.R. (2021). Wool Keratin Hydrolysates for Bioactive Additives Preparation. Materials.

[42] Liu H., Fiorani F., Jäck O., Colombi T., Nagel K.A., Weih M. (2021). Shoot and Root Traits Underlying Genotypic Variation in Early Vigor and Nutrient Accumulation in Spring Wheat Grown in High-Latitude Light Conditions. Plants.

[43] Yetilmezsoy K., Ilhan F., Kıyan E. (2025). Utilization of Non-Composted Human Hair Hydrolysate as a Natural and Nutrient-Rich Liquid Fertilizer for Sustainable Agro-Applications and Bio-Waste Management. Sustainability.

[44] Kaniszewski S., Kowalski A., Dysko J., Agati G. (2021). Application of a Combined Transmittance/Fluorescence Leaf Clip Sensor for the Nondestructive Determination of Nitrogen Status in White Cabbage Plants. Sensors.

[45] Zuzunaga-Rosas J., Boscaiu M., Vicente O. (2024). Agroindustrial By-Products as a Source of Biostimulants Enhancing Responses to Abiotic Stress of Horticultural Crops. Int. J. Mol. Sci..

[46] Goncharuk E.A., Zagoskina N.V. (2023). Heavy Metals, Their Phytotoxicity, and the Role of Phenolic Antioxidants in Plant Stress Responses with Focus on Cadmium: Review. Molecules.

[47] Hassani S.B., Latifi M., Aliniaeifard S., Sohrabi Bonab S., Nasiri Almanghadim N., Jafari S., Mohebbifar E., Ahangir A., Seifikalhor M., Rezadoost H. (2023). Response to Cadmium Toxicity: Orchestration of Polyamines and microRNAs in Maize Plant. Plants.

